# Emerging preclinical evidence does not support broad use of hydroxychloroquine in COVID-19 patients

**DOI:** 10.1038/s41467-020-17907-w

**Published:** 2020-08-26

**Authors:** S. G. P. Funnell, W. E. Dowling, C. Muñoz-Fontela, P.-S. Gsell, D. E. Ingber, G. A. Hamilton, L. Delang, J. Rocha-Pereira, S. Kaptein, K. H. Dallmeier, J. Neyts, K. Rosenke, E. de Wit, H. Feldmann, P. Maisonnasse, R. Le Grand, M. B. Frieman, C. M. Coleman

**Affiliations:** 1 National Infection Service, Public Health England, Porton Down, Manor Farm Road, Salisbury, Wiltshire SP40JG UK; 2 Coalition for Epidemic Preparedness Innovations, 1901 Pennsylvania Avenue, NW, Suite 1003, Washington, DC 20006 USA; 3 Bernhard Nocht Institute for Tropical Medicine, Bernhard Nocht Strasse. 74, 20359 Hamburg, Germany; 4 World Health Organisation, Avenue Appia, 1211, Geneva, Switzerland; 5 Wyss Institute for Biologically Inspired Engineering, Harvard University, CLSB5, 3 Blackfan Circle, Boston, MA 02115 USA; 6 Emulate Inc., 27 Drydock Avenue, 5th Floor, Boston, MA 02210 USA; 7 Laboratory of Virology and Chemotherapy, Department of Microbiology, Immunology and Transplantation, Rega Institute, Global Virus Network, KU Leuven, 3000 Leuven, Belgium; 8 Laboratory of Virology, Division of Intramural Research, National Institute of Allergy and Infectious Diseases, National Institute of Health, Hamilton, 59840 MT USA; 9 Université Paris-Saclay, Inserm, CEA, Center for Immunology of Viral, Auto-immune, Hematological and Bacterial diseases » (IMVA-HB/IDMIT), Fontenay-aux-Roses & Le Kremlin-Bicêtre, 92265 France; 10 Department of Microbiology and Immunology, University of Maryland School of Medicine, Baltimore, MD 21201 USA; 11 School of Life Sciences, University of Nottingham, Queen’s Medical Centre, Nottingham, NG7 2UH UK

**Keywords:** Animal disease models, Respiratory system models, SARS-CoV-2, Preclinical research

## Abstract

There is an urgent need for drugs, therapies and vaccines to be available to protect the human population against COVID-19. One of the first approaches taken in the COVID-19 global response was to consider repurposing licensed drugs. This commentary highlights an extraordinary international collaborative effort of independent researchers who have recently all come to the same conclusion—that chloroquine or hydroxchloroquine are unlikely to provide clinical benefit against COVID-19.

As part of the World Health Organisation’s (WHO) Research and Development Blueprint response to the COVID-19 outbreak, an ad hoc working group of scientists was convened in February 2020 to encourage data sharing, to help avoid repetition of effort and to encourage reduction, refinement and replacement in animal experimentation. This WHO-led effort has resulted in an unprecedented level of international data sharing and collaboration across more than 80 countries. In the course of the 11th meeting of the WHO ad hoc working group on COVID-19 infection modelling (7th May 2020), several groups reported recent findings using different SARS-CoV-2 infection models with chloroquine (CQ) and/or hydroxychloroquine (HCQ).

HCQ is a less toxic form of CQ, as it carries an additional hydroxyl group. Both CQ and HCQ are used to prevent and treat malaria in endemic areas of the world. Due to their anti-inflammatory properties, CQ and HCQ are also used to treat autoimmune disorders such as lupus erythematosus and rheumatoid arthritis. During the Ebola virus disease (EVD) epidemic in West Africa, these drugs caught the attention of the media after several reports indicated a possible association between CQ treatment and amelioration of EVD symptomatology. Amidst the COVID-19 pandemic, these drugs have again been brought into the spotlight as putative therapeutics against SARS-CoV-2 and several clinical trials have been conducted or are underway using them.

This Comment highlights five sets of important recent findings from several independent groups relating to the preclinical efficacy of CQ and HCQ, raising doubts about the putative benefit of these drugs to treat COVID-19.

## Advantages and disadvantages of different preclinical models

Although mouse infection models permit greater numbers to be used and allow a broad range of analysis due to general murine reagent availability, mice are innately resistant to SARS-CoV-2. As a result, either the virus needs to be adapted to be more infective in mice or there needs to be a change in the natural state of mice to allow infection. This is possible via either adapting mice to express the human ACE2 virus receptor or in some way reducing their natural immunity.

Hamsters are naturally susceptible to SARS-CoV-2 infection and demonstrate mild to moderate disease. Unlike mice, however, there is currently only a limited catalogue of reagents available to study them. Ferrets are also naturally susceptible but display only mild disease as in many human cases.

Non-human primates are the closest means of modelling human infection as their innate and adaptive immunity, as well as their physiology and anatomy, are close to man. Like hamsters and ferrets, they are naturally susceptible but generally develop a mild form of disease, which is cleared within a week or two unless they are elderly. The difficulty with non-human primate research is that there are considerable costs, ethical constraints and limited availability due to high demand.

More complex in vitro systems have now also been developed such as the Emulate and MucilAir^TM^ systems. These microfluidic systems can use human tissue cells (avoiding cross-species differences), have excellent physiological and morphological properties and permit larger numbers to be used in each study in a smaller high containment laboratory footprint. The disadvantages of these are that there are only a few laboratories that have the specialist technical resources to use them although this is likely to change in the foreseeable future as these systems become commercially available.

## Outcomes in simple in vitro models using CQ and HCQ against SARS-CoV-2

CQ and HCQ can be considered as host-targeting antivirals, this is in contrast to specific directly acting antivirals such as those available to treat infections with herpesviruses, HIV, HBV, HCV and influenza. Whereas CQ and HCQ inhibit SARS-CoV, MERS-CoV and SARS-CoV-2 in simple cell culture systems like Vero cells^1^ (IC_50_ ~3–10 μM) they do not appear to be effective at inhibiting virus replication in other more complex preclinical human and animal models.

## Recent outcomes in complex in vitro human models

In recent studies, human lung airway-chips have been shown to recapitulate clinical responses to cigarette smoke and drugs^2,3^, and to model human lung responses to infection by viruses, including influenza^4^.

Multiple FDA approved drugs that had been shown to inhibit SARS-CoV-2 in Vero cells by different groups, including CQ, were tested using SARS-CoV-2 pseudoviruses (lentivirus particles pseudotyped with the SARS-CoV-2 spike protein) in the Emulate human lung-chips^5^. When flowing the drugs through lung-chip devices, at a clinically relevant dose (the reported human Cmax) to mimic how drugs are delivered to organs in our bodies, CQ did not produce statistically significant inhibition of replication of the SARS-CoV-2 Spike pseudotyped viruses.

Meanwhile in France, a research team at Inserm have developed another complex human in vitro model system (MucilAir^TM^), which is developed from primary nasal or bronchial cells differentiated and cultivated under an air/liquid interphase. In alignment with the findings by the Wyss Institute, Inserm conclude that HCQ does not significantly inhibit SARS-CoV-2 infection^6^ in their human respiratory tissue model.

## Previous outcomes in mice using SARS-CoV

The efficacy of CQ has previously been tested in vivo using a mouse adapted SARS-CoV (MA15) challenge to evaluate anti-viral efficacy.

In these experiments, 10-week-old Balb/c mice were intraperitoneally (IP) injected at 40 mg/kg/day or 80 mg/kg/day starting 1 day before infection and then daily during the course of the infection. Mice were intranasally (IN) inoculated with 1E + 05 pfu/mouse MA15 and their outcome followed for 4 days. MA15 infected mice that were IP injected with carrier lost 20% of their body weight and demonstrated clinical illness (ruffled fur and laboured breathing) over 4 days of the experiment while those given 80 mg/kg did not lose weight.

At day 2 and 4 post infection (dpi), minimal inflammation in the lungs of infected and drug treated mice were observed while vehicle treated mice had significant lung inflammation. Virus lung titre was also analysed 2 and 4 dpi and contrary to the clinical and histological symptoms, there was no effect detected. This led to the hypothesis that the in vitro effects of CQ on MA15 and other coronaviruses was affecting entry and lipid membrane alterations, while in vivo the main effect was anti-inflammatory rather than anti-viral. This demonstrated that while there are features of infection that are altered by CQ treatment, it did not diminish viral replication in this model^7^.

## Outcomes in hamster efficacy studies using SARS-CoV-2

Several laboratories have recently found that hamsters are susceptible to infection with low passage SARS-CoV-2^8,9^. In studies conducted at KU Leuven, Belgium, the effect of HCQ either alone or in combination with azithromycin was explored in the hamster SARS-CoV-2 infection model^10^. Wild-type hamsters were IN infected with 2E + 06 tissue culture infectious dose 50 (TCID_50_) of a Belgian SARS-CoV-2 isolate. Replicating virus was detected in the lungs as soon as one day after inoculation and titres increased rapidly over the next days, reaching a plateau at 4 dpi.

HCQ was administered to infected hamsters at a dose of 50 mg/kg/day given by IP injection starting 1 h before infection and continued once daily until the end of the experiment. This resulted in insignificant reductions of detectable viral RNA and infectious virus titres in the lungs of infected animals (*n* = 4) at 4 dpi.

Another group of hamsters received the same dose of HCQ treatment in addition to 10 mg/kg/day of azithromycin, given orally once daily with a similar schedule to that of HCQ. This combined treatment resulted in 0.5 log_10_ higher viral RNA levels when compared to the vehicle group, and 0.3 log_10_ reduction in infectious virus titres in the lungs of the infected hamsters.

The selected doses of HCQ and azithromycin were based on literature and are expected to result in sufficient exposure of either drug. Although these are data from a pilot experiment and currently have no accompanying PK data, these results suggest that HCQ alone or in combination with azithromycin has not demonstrated a protective effect from viral replication in the lungs of infected hamsters. The SARS-CoV-2 hamster infection model has been verified for anti-viral studies with other agents.

Meanwhile, independent studies were also being conducted at the NIAID Rocky Mountain Laboratories (RML) using the Syrian hamster disease model. RML tested the efficacy of HCQ prophylaxis and treatment using a low (6.5 mg/kg) and high (50 mg/kg) dose regimen in comparison to control groups treated with vehicle only^11^. HCQ delivery was via IP injection of the drug and SARS-CoV-2 infection was performed by the IN route with a dose of 1E+04 TCID_50_ per hamster. The prophylactic treatment was performed once, 24 hours prior to infection. The therapeutic treatment started 1 h after SARS-CoV-2 infection and continued for 3 days.

The RML experiments revealed no significant difference in disease manifestation and progression nor in virus replication and shedding among vehicle treated control and HCQ treated groups for both arms of the study, prophylaxis and treatment.

## Outcomes in a Cynomolgus macaque model of SARS-CoV-2

The outcomes in human organ cultures and hamsters are now also supported by a preclinical assessment of HCQ in a SARS-CoV-2 infection model developed in cynomolgus macaques at Inserm^6^.

One control group of 8 animals and five treated groups of 4 to 5 animals were challenged with 1.0E + 06 pfu by a combination of the intranasal and intra-tracheal routes. Animals were treated daily by gavage with either vehicle (water) or HCQ. Two groups received a high dose regimen of HCQ (loading dose 90 mg/kg 1 dpi and daily maintenance dose 45 mg/kg), for a total of 10 days. One group also received AZTH (36 mg/kg at 1 dpi, then 18 mg/kg). Two groups received a low dose regimen of HCQ (loading dose 30 mg/kg and daily maintenance dose 15 mg/kg) for 12 days, starting either at D1 or D5 post-exposure. A Pre-exposure prophylaxis (PrEP) group received a loading dose of 30 mg/kg 7 days before challenge, followed by daily doses of 15 mg/kg for 4 days and then 45 mg/kg from 3 days before until 6 dpi. No significant difference was observed between the drug-treated and the vehicle-treated animals either in terms of viral loads in the respiratory tract, lesions observed by chest CT or clinical signs. The results of these studies demonstrate no significant anti-viral or clinical benefit of the HCQ when given as a PrEP or after infection, at several doses and with or without azithromycin.

The results of these studies demonstrate no significant anti-viral or clinical benefit of the HCQ when given at several doses and time-points.

The studies at Inserm also analysed the pharmacokinetics and biodistribution of HCQ in cynomolgus macaques and confirmed strong accumulation in the lung tissue, with concentrations highly superior to the EC_50_ estimated from in vitro models. These data show that the lack of efficacy of HCQ is not related to low penetration of HCQ in the relevant tissues targeted by the virus. When HCQ was given either before or after infection, no protection was observed in cynomolgus macaques.

Although the cynomolgus macaque model of COVID-19 does not allow the exploration of anti-inflammatory properties of HCQ in late severe cases of the disease, these data demonstrate the absence of anti-viral effect of HCQ.

## Outcomes in a Rhesus macaque model of SARS-CoV-2

The effect of HCQ prophylaxis and treatment has also recently been tested at RML using a SARS-CoV-2 infection model in rhesus macaques^11,12^. In the first study component, ten healthy rhesus macaques were randomly divided into vehicle control and HCQ prophylaxis groups (*n* = 5 per group). Animals were treated using a gastric tube with either vehicle (PBS) or HCQ (6.5 mg/kg in PBS) three times 1 week apart. In the second study component, ten healthy rhesus macaques were randomly divided into vehicle control and HCQ treatment groups (*n* = 5 per group). Animals were treated using a gastric tube with either vehicle (PBS) or HCQ (6.5 mg/kg in PBS) starting 12 h post infection followed by one treatment each day for 6 days. The animals in the prophylaxis groups were infected following HCQ or vehicle application and those in the treatment group prior to HCQ or vehicle treatment with SARS-CoV-2, total dose 2.8E + 06 TCID_50_ by a combination of four routes (intra-tracheal, oral, intranasal and ocular). Animals were euthanized on day 7 post infection and assessed for virology, immunology and pathology. Animals in both drug-treated and vehicle-treated groups and both prophylaxis and treatment regimens developed similar mild to moderate disease with indistinguishable SARS-CoV-2 replication and shedding in the lower and upper respiratory tract. In conclusion, neither HCQ prophylaxis nor treatment showed any benefit in this SARS-CoV-2 infection model.

## Conclusion and recommendation

In simple Vero cell assays, CQ and HCQ appear to demonstrate anti-viral properties. However, these in vitro effects are not seen in more complex and life-like models of infection such as organ-on-chips, which use human respiratory cells. In addition, no significant therapeutic benefit derived from HCQ was observed in SARS-CoV-2 infection model studies in hamsters and non-human primates. These consistent findings have been observed in four independent laboratories.

In summary, these data do not support the broad use of HCQ to treat or prevent SARS-CoV-2 disease. These preclinical study results appear to align with some clinical studies^13^ and the recently unveiled outcome from the RECOVERY trial in the UK (http://www.ox.ac.uk/news/2020-06-05-no-clinical-benefit-use-hydroxychloroquine-hospitalised-patients-covid-19-0). They also align with the FDA’s recent decision on the 15/June/2020 to remove the emergency use authorisation it granted on the 28/March/2020 (https://www.fda.gov/news-events/press-announcements/coronavirus-covid-19-update-fda-revokes-emergency-use-authorisation-chloroquine-and).

All animal work described by each contributor of this paper was approved by their respective institutions and complied with national animal care and use guidelines.
